# Non-functional thyroid cystadenoma in three boxer dogs

**DOI:** 10.1186/s12917-019-1948-z

**Published:** 2019-07-05

**Authors:** Marie-Pauline Maurin, Dan Davies, Hanne Jahns, Robert E. Shiel, Carmel T. Mooney

**Affiliations:** School of Veterinary Medicine, University College Dublin, Dublin, Ireland

**Keywords:** Thyroid neoplasm, Boxer, Thyroid cystic adenoma, Thyroid cyst, Cervical mass

## Abstract

**Background:**

Thyroid neoplasia is a common endocrine neoplasm in dogs. The boxer is one of the reported breeds predisposed to malignant thyroid neoplasia. However, the association between thyroid neoplasia, malignancy and breed should be considered with caution.

**Cases presentation:**

This article describes the presentation, clinical pathological findings, computed tomographic (CT) imaging findings and histopathological features of benign cystic thyroid tumour (cystadenoma) diagnosed in three boxers. These three dogs were presented for investigation of unilateral (*n* = 2) or bilateral (*n* = 1) cervical masses with no associated clinical signs of thyroid dysfunction. In each case, post-contrast CT scan identified a large, lateralised, non-invasive, well-defined homogeneous cystic structure with a hyperattenuating contrast-enhancing capsule of suspected thyroid origin displacing the surrounding cervical tissues. Ultrasound-guided fine needle aspiration of the cysts yielded fluid with a high thyroxine concentration in each case. Histopathology was consistent with thyroid cystadenoma in all cases. One dog was concurrently diagnosed with oral melanoma and euthanased. Two dogs underwent surgical excision with one lost to follow-up after 36 months and the other euthanased after 16 months following diagnosis of mast cell tumour.

**Conclusions:**

To the authors’ knowledge, this is the first detailed report of non-functional benign thyroid cystadenoma in dogs and provides relevant information about case management for this type of tumour. The presence of a large cystic structure associated with benign non-functional thyroid neoplasia may be a condition to which boxer dogs are predisposed.

## Background

Thyroid tumours account for 1.1 to 3.8% of all neoplasms and approximately 10–15% of all head neoplasms in dogs [1–5]. In some studies, boxers, beagles, golden retrievers and Siberian huskies were more commonly affected compared to other breeds [2, 4–8]. However, another recent study found no association between breed and development of thyroid neoplasia [1]. Thyroid carcinomas are more frequently diagnosed *antemortem* than thyroid adenomas [1, 4–6, 15], which are often discovered serendipitously during physical examination or at necropsy [5, 8]. Dogs with thyroid carcinomas have evidence of metastasis at the time of diagnosis in from 16 to approximately 60% of case [11, 14, 16]. Carcinomas are generally large in size with rapid growth and aggressive biological behaviour. They can extend into or around the wall of the trachea, cervical muscles, oesophagus, larynx, nerves and vessels [12]. Early invasion of the cranial and caudal thyroid veins with formation of tumour cell thrombi can lead to pulmonary metastases even before involvement of the retropharyngeal and caudal cervical lymph nodes [7, 11]. The majority of dogs with thyroid tumours are euthyroid, although both hyperthyroidism and hypothyroidism have been described [9–11]. In a recent large study of canine thyroid carcinoma, 12 of 57 (21%) cases where thyroid function was investigated were hyperthyroid [10]. Up to 30% of dogs are described as hypothyroid secondary to destruction of the normal thyroid parenchyma [12]. In accord with this, one experimental study of Beagles suggested that hypothyroidism may be a pre-existing condition [13]. However, in many older studies the diagnosis of hypothyroidism is questionable. In a recent study, only 2 of 57 (3.5%) of cases were truly hypothyroid, although such a diagnosis could not be excluded in approximately 30% of cases [10].

Marked rapid enlargement of the thyroid gland could also be caused by cysts. Thyroid cysts are fluid filled cavities, in dogs mainly as thyroid cystadenomas [17]. Cysts can also be observed with malignant thyroid tumours, namely papillary thyroid carcinomas, which are rarely reported in dogs [15, 17]. Although cystadenomas are occasionally mentioned in large case series of thyroid neoplasia in dogs, or as incidental post-mortem findings, detailed clinical features of these cases have not been described [1, 5, 7, 18]. Two individual case reports described thyroid cystadenoma with concurrent hyperthyroidism in a German shepherd dog and an English springer spaniel [19, 20].

The current case report describes the clinical signs, diagnosis and treatment of thyroid cystadenomas in three boxer dogs that presented with enlarging masses on the ventral neck. The presence of hyperthyroidism was not identified in any case. Furthermore, the measurement of the thyroxine (T4) concentration in the cystic fluid allowed rapid confirmation of the thyroidal origin of the mass in each case.

## Case presentations

Between 2012 and 2018, three boxer dogs were independently evaluated at University College Dublin (UCD) Veterinary Hospital for investigation of ventral cervical masses (Table 1). The masses were present for a period ranging from six weeks to six months prior to referral. Episodes of regurgitation after excitement or strenuous exercise were also reported in case 1, and hypersalivation in case 3. In cases 1 and 2, the primary veterinary surgeon drained fluid from the mass by percutaneous fine needle aspiration prior to referral; the swelling recurred within a few weeks in both cases.

**Table 1:** Signalment, histopathologic diagnosis and outcome in three dogs with thyroid cystadenoma

Cases	Gender	Age (years)	Diagnosis	Outcome
1	Male castrated	8	Thyroid cystadenoma (Right lobe)	Last follow-up 36 months after surgical removal
2	Male castrated	8	Thyroid cystadenoma (Left lobe)	Euthanased 16 months after surgical removal
3	Female neutered	10	Thyroid cystadenoma (Left lobe)	Euthanased

At presentation, palpation of the ventral neck revealed a 3 to 5 cm diameter, mobile, well-circumscribed, non-painful, ovoid, subcutaneous mass in close proximity to the trachea in each case. Mandibular and pre-scapular lymph node palpation revealed no abnormalities. Cardio-thoracic auscultation and abdominal palpation were unremarkable in all cases. No dermatological or neurological abnormalities were noted in any case.

There were no haematological abnormalities in any case (Table 2). Serum biochemistry identified hypercholesterolaemia in case 3. Otherwise only mildly increased liver enzyme activities of no clinical significance were noted (Table 2). Total T4 and canine thyroid-stimulating hormone (cTSH) concentrations were measured in cases 2 and 3 (Table 2). Results were most consistent with non-thyroidal illness although additional testing (e.g. measurement of free T4 concentration or thyroglobulin autoantibody status) was not performed. In case 2, the cTSH concentration approached the upper limit of the reference interval at the time of initial presentation. Repeat measurements of T4 and cTSH were recommended and performed six weeks after surgical treatment.

**Table 2:** Haematological and biochemical findings in three dogs with thyroid cystadenoma

	Case 1 (Reference intervals)	Case 2 (Reference intervals)	Case 3 (Reference intervals)
Haematocrit	0.48 (0.37–0.55 L/L)	0.49 (0.37–0.55 L/L)	0.46 (0.37–0.55 L/L)
Haemoglobin	170 (120–180 g/L)	164 (120–180 g/L)	156 (120–180 g/L)
Red Blood Cells	7.10 (5.5–8.5 × 10^12^/L)	7.09 (5.5–8.5 × 10^12^/L)	6.24 (5.5–8.5 × 10^12^/L)
MCHC	352 (310–362 g/L)	333 (315–370 g/L)	340 (310–362 g/L)
MCV	68.1 (60–77 fL)	68.2 (60–80 fL)	73.5 (60–77 fL)
Platelet count	261 (150–500 × 10^9^/L)	216 (160–500 × 10^9^/L)	339 (150–500 × 10^9^/L)
White Blood Cells	7.31 (6–17 × 10^9^/L)	7.40 (6–15 × 10^9^/L)	6.81 (6–17 × 10^9^/L)
Neutrophil count	4.56 (3–11.5 × 10^9^/L)	5.18 (3–11.5 × 10^9^/L)	3.13 (3–11.5 × 10^9^/L)
Lymphocyte count	2.07 (1–3.6 × 10^9^/L)	1.55 (1–4.8 × 10^9^/L)	2.66 (1–3.6 × 10^9^/L)
Monocyte count	0.37 (0–1.35 × 109/L)	0.52 (0–1.30 × 109/L)	0.61 (0–1.35 × 109/L)
Eosinophil count	0.26 (0–1.47 × 109/L)	0.07 (0–1.25 × 109/L)	0.41 (0–1.47 × 109/L)
Smear	NAD	NAD	NAD
Total protein	69.7 (54–71 g/L)	62.0 (54–77 g/L)	69.7 (54–71 g/L)
Urea	4.5 (3.6–8.6 mmol/L)	4.9 (2.0–9.0 mmol/L)	7.2 (3.6–8.6 mmol/L)
ALT	50 (0–36 U/L)	67 (0–25 U/L)	61 (0–36 U/L)
GGT	0 (0–16 U/L)	n/p	2 (0–16 U/L)
CK	106 (0–122 U/L)	77 (0–190 U/L)	76 (0–122 U/L)
Amylase	974 (400–1300 U/L)	1063 (0–1800 U/L)	1039 (400–1300 U/L)
Cholesterol	5.97 (3.2–6.5 mmol/L)	6.50 (3.8–7.0 mmol/L)	8.87 (3.2–6.5 mmol/L)
Phosphate	1.37 (0.8–1.8 mmol/L)	1.10 (0.8–1.6 mmol/L)	1.30 (0.8–1.8 mmol/L)
Sodium	150.4 (137–151 mmol/L)	148.8 (139–154 mmol/L)	151.2 (137–151 mmol/L)
Chloride	109.6 (105–117 mmol/L)	109.0 (99–125 mmol/L)	111.3 (105–117 mmol/L)
Triglycerides	0.39 (0.11–1.69 mmol/L)	0.42 (0.45–1.90 mmol/L)	0.73 (0.11–1.69 mmol/L)
Albumin	31.8 (25–38 g/L)	32.0 (26–40 g/L)	33.0 (25–38 g/L)
Creatinine	94 (20–120 umol/L)	95 (40–106 umol/L)	75 (20–120 umol/L)
ALP	92 (0–82 U/L)	44 (0–25 U/L)	129 (0–82 U/L)
GLDH	3 (0–16 U/L)	4 (0–10 U/L)	6 (0–16 U/L)
Lipase	28 (0–130 U/L)	36 (0–150 U/L)	32 (0–130 U/L)
Glucose	5.4 (3–6.5 mmol/L)	4.8 (2.0–5.5 mmol/L)	6.6 (3–6.5 mmol/L)
Total Bilirubin	5.2 (0.9–10 umol/L)	1 (0–9.0 umol/L)	3.8 (0.9–10 umol/L)
Calcium (total)	2.60 (2.3–3 mmol/L)	2.64 (2–3 mmol/L)	2.60 (2.3–3 mmol/L)
Potassium	4.44 (3.7–5.8 mmol/L)	4.73 (3.5–6.0 mmol/L)	3.79 (3.7–5.8 mmol/L)
Globulin	37.9 (28–42 g/L)	30.0 (20–47 g/L)	36.7 (28–42 g/L)
AST	25 (0–37 U/L)	n/p	21 (0–37 U/L)
Total T4	n/p	14.9 (15–60 nmol/L)	12.1 (15–60 nmol/L)
TSH	n/p	0.643 (< 0.68 ng/mL)	0.275 (< 0.68 ng/mL)
T4 in cystic fluid	> 193.0 nmol/L	111.0 nmol/L	> 193.0 nmol/L

Total T4/TSH serum concentration and T4 fluid concentration

n/p: not performed

NAD: no abnormalities detected

T4, thyroxine

TSH, thyroid stimulating hormone

Cervical ultrasonography confirmed an ovoid, cystic lesion in the region of the right thyroid lobe in case 1 and of the left lobe in case 2. In case 3, a large cystic structure was localised in the left thyroid lobe and several small cystic structures in the right thyroid lobe. Ultrasound-guided aspiration of the cystic lesions was performed in all cases. In cases 1 and 2, cytologic analysis of the fluid contained in the large cystic lesions was non-diagnostic, revealing proteinaceous fluid with mild cell necrosis. In case 3, fine needle aspirate of the heterogeneous part of the left thyroid lobe was performed and cytological analysis revealed abundant cellular debris, small numbers of red blood cells (RBC) and very large cells up to 10 times RBC diameter with eccentrically displaced large nuclei with predominantly cytoplasmic area filled with light-to-dark blue material with frequent coarse dark-blue granules. Occasionally, these cells occured in small clusters with indistinct cell borders. No significant criteria of malignancy were found. These features were considered consistent with thyroid gland adenoma with marked cell necrosis; however, well-differentiated carcinoma could not be excluded. Some of the aspirated fluid was placed in plain tubes for T4 measurement using the same assay as for serum samples (Immulite 2000 Canine Total T4, Siemens Healthcare Diagnostics using an Immulite 2000 Analyzer). Concentrations were markedly increased (compared to the serum reference interval) in each case, confirming thyroidal origin (Table 2).

**Fig. 1:**
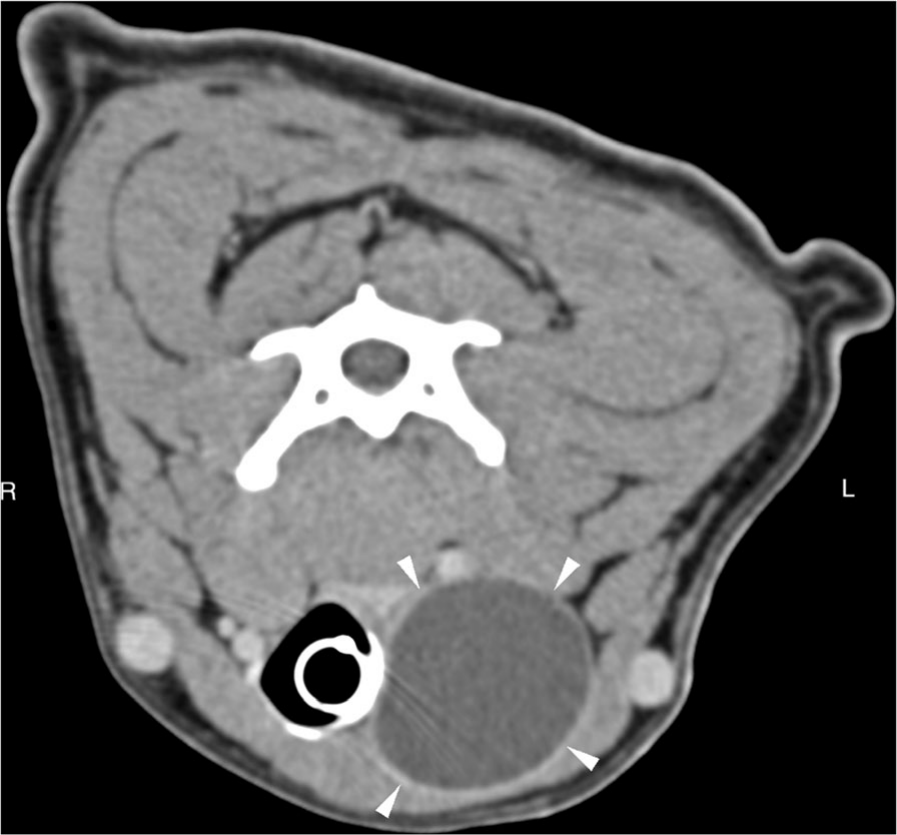
Case 2: Transverse plane CT reconstruction at the level of the mid-body of the third cervical vertebra in soft tissue window, post intravenous contrast administration. A well-defined large (4.3 cm length × 3.5 cm height × 3.3 cm width), ovoid and fluid attenuating structure with a thin peripherally contrast-enhancing margin is located to the left lateral aspect of the trachea (white arrowheads). An endotracheal tube is located within the tracheal lumen

**Fig. 2:**
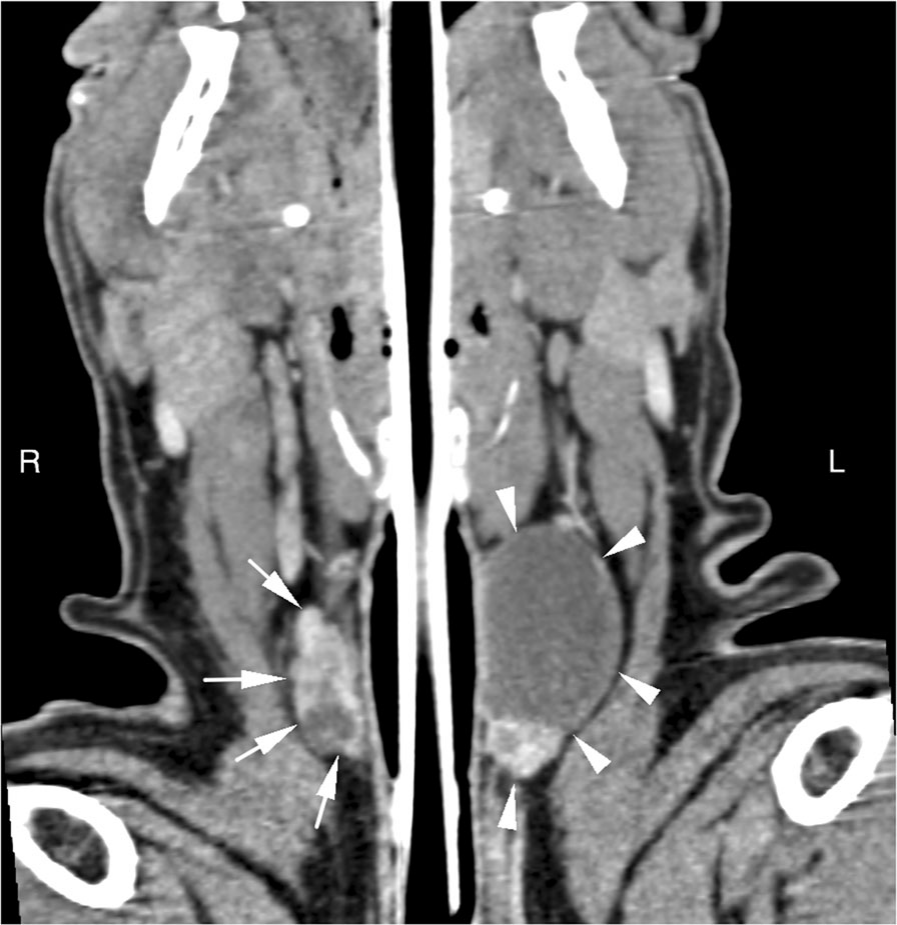
Case 3: Dorsal plane CT reconstruction at the level of the thyroid glands in soft tissue window, post intravenous contrast administration. Cranial is at the top of the image. The right lobe of the thyroid gland (white arrows) is heterogeneously contrast enhancing and contains multiple poorly defined fluid attenuating structures surrounded by contrast enhancing tissue. The caudal aspect of the left lobe of the thyroid gland (white arrowheads) is similarly heterogeneously contrast enhancing. The cranial aspect of the left lobe of the thyroid gland is enlarged (3.6 cm length × 2.2 cm height × 2.5 cm width), and contains an ovoid fluid attenuating structure surrounded by a thin margin of peripheral contrast enhancement. An endotracheal tube is located within the tracheal lumen

Plain computed tomography (CT) of the neck and chest were performed for staging purpose in all cases and demonstrated ovoid, well-defined, homogeneous fluid attenuating structures within the ventral neck at the level of the third cervical vertebra on the right side in case 1, and left side in cases 2 and 3. The structures measured 3.7 × 2.7 cm in case 1, 3.1 × 4.2 cm in case 2, and 4.8 × 2.7 cm in case 3. In all cases, the mass partially displaced the trachea and surrounding structures to the contralateral side. Post-contrast CT identified a hyperattenuating contrast-enhancing margin in all cases with no evidence of invasion into surrounding tissues. No other structure compatible with thyroid tissue could be identified on the ipsilateral side of the trachea in case 1 and 2. The contralateral thyroid lobe showed no enlargement or other abnormality in case 1 or 2 (Fig. 1). In case 3, the right thyroid lobe was enlarged and lobulated with multiple ovoid, poorly-defined, hypoattenuating nodules separated by thin bands of hyperattenuating contrast-enhancing tissue. The large cystic structure was at the level of the cranial pole of the left lobe, and the caudal pole was heterogeneously contrast enhancing similar to the right lobe (Fig. 2). There was no evidence of enlarged lymph nodes or pulmonary metastasis identified on the CT images.

**Fig. 3:**
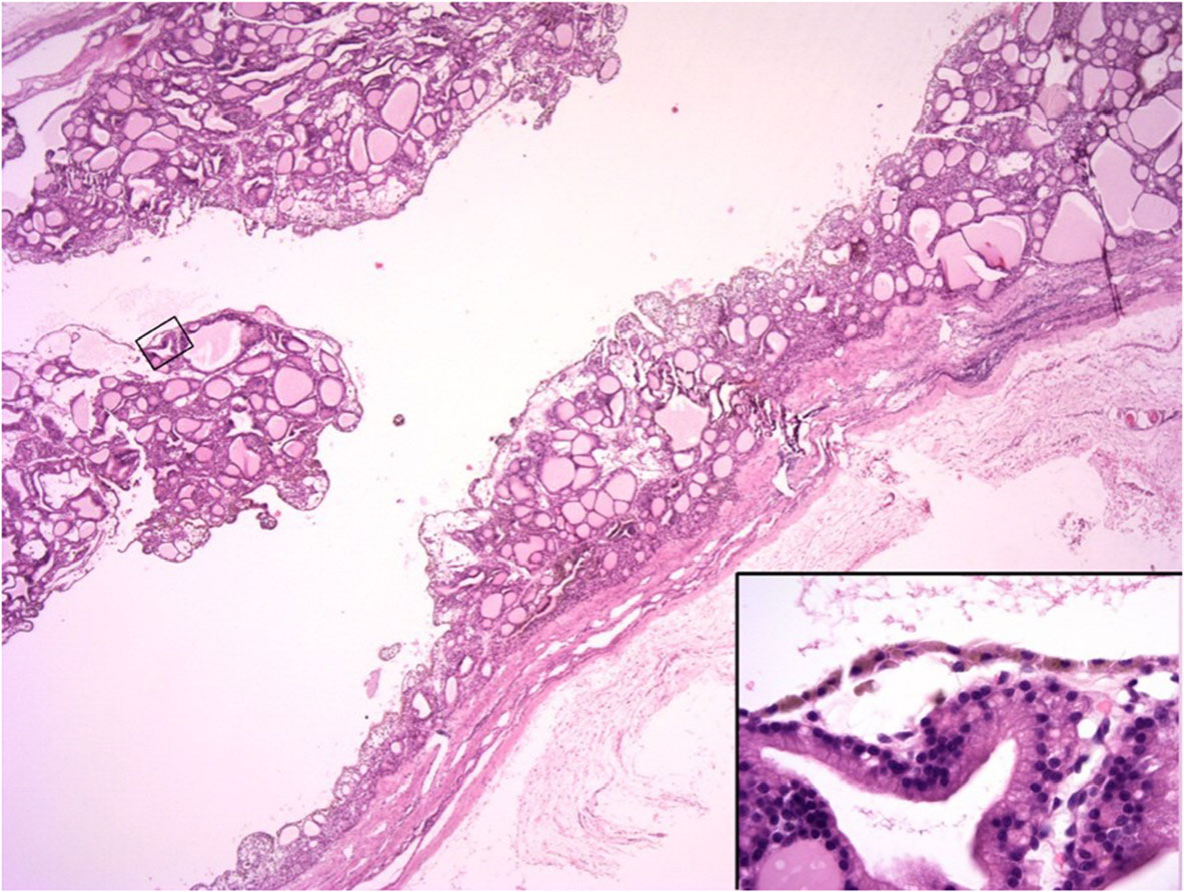
Case 1: Thyroid cystadenoma. The large cystic cavity is lined by cuboidal epithelial cells and contains scant proteinaceous fluid and cell debris. Tumour cells forming either follicles or solid nests surround the cyst and project into the lumen. H&E 20x and inset 400x magnification

**Fig. 4:**
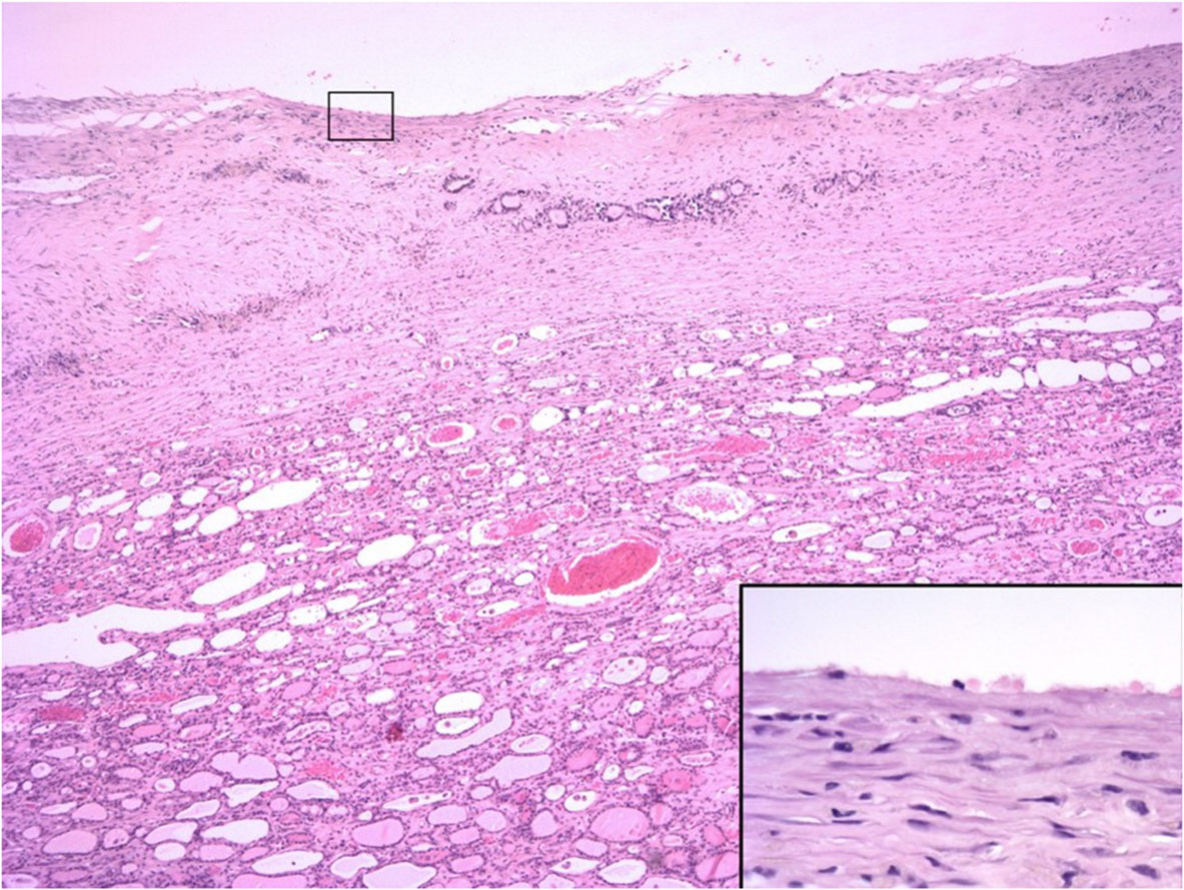
Case 2: Thyroid cystadenoma. The cyst is lined by a thick wall composed of collagen rich fibrous tissue with a few trapped follicles. The remaining thyroid tissue is compressed. H&E 40x and inset 400x magnification

Surgical excision of the thyroid lobe containing the cystic structure was performed in cases 1 and 2 via routine ventral midline incision of the neck. In both cases similar intraoperative findings were observed and comparable techniques were performed. The cystic structure was moderately adherent and gently isolated and separated from the surrounding tissues by blunt dissection. It was detached caudally, then reflected rostrally and the main vascularisation, which was found cranially, was ligated and the cystic structure removed. In both cases the cystic structure was removed intact and no perforation occurred during dissection. Surgery and anaesthetic recovery were uneventful. Excised thyroid tissue of case 1 and 2 was submitted for histopathology and in case 3, the entire thyroid gland was submitted following necropsy. Histopathology revealed similar morphological features in all three cases, which consisted of large cystic cavities containing proteinaceous fluid, erythrocytes and cell debris lined by cuboidal cells which were often flattened and attenuated, or occasionally lost (Fig. 3). When neoplastic cuboidal lining cells were lost, the cyst was lined by thick collagen-rich fibrous tissue with a few trapped follicles (Fig. 4). In all samples examined including both thyroid lobes of case 3, the solid thyroid tissue adjoining the cysts was composed of cuboidal neoplastic cells with round-to-oval, normochromatic nuclei forming variably-sized follicles or small, solid nests. The adjacent thyroid tissue was compressed. Based on the above features, a diagnosis of thyroid cystadenoma was made in all three cases.

Cases 1 and 2 were discharged 48 and 72 h postoperatively, with follow-up times of 36 and 16 months, respectively. No surgical complications were reported. In case 2, as recommended, thyroid function was re-assessed six weeks after surgery. The results indicated decreased total T4 (8.22 (15–60) nmol/L) and increased cTSH (2.38 (< 0.68) ng/ml) concentrations. Primary hypothyroidism was diagnosed and levothyroxine (Soloxine; Virbac) prescribed. Four months after surgery, an intermediate grade Patnaik, high-grade Kiupel dermal scrotal mast cell tumour was diagnosed. The tumour was surgically excised completely and adjunctive masitinib (Masivet, AB Science) was provided. Metastasis of the mast cell tumour to the abdominal lymph nodes occurred a year later while the dog was still receiving chemotherapy, and euthanasia was elected. A rapidly progressive oral amelanotic melanoma was also diagnosed in case 3 at the same time as presentation for the cervical mass. The dog was euthanased and post-mortem examination performed.

**Fig. 5:**
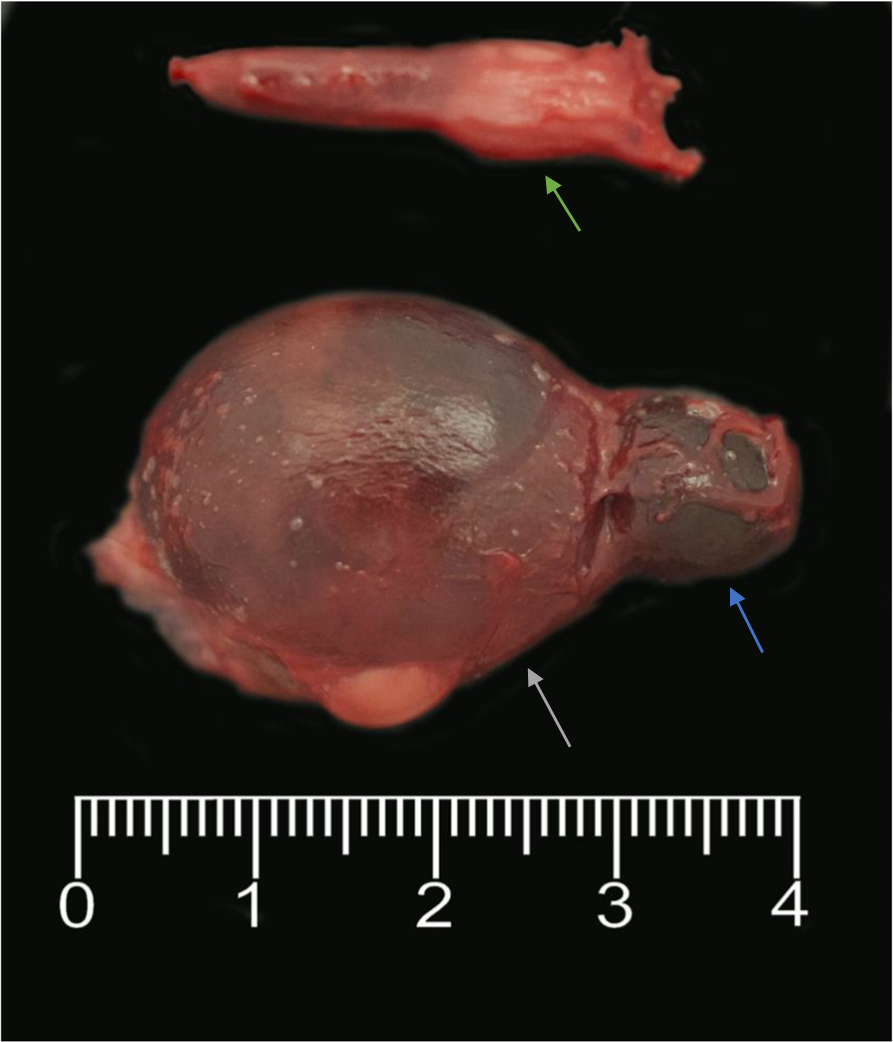
Boxer (U243968); thyroid glands; The left thyroid was 1 × 0.5 cm in size, the right was enlarged, with two soft cystic lesions (blue arrow =1.5 × 1 cm, green arrow = 3.5 × 2.5 cm). Marked parathyroid hyperplasia is also observed (grey arrow), likely due to concurrent advanced chronic renal disease diagnosed in this case

In order to further investigate a possible predisposition for thyroid cystadenoma in the boxer breed, the database of all the samples (internal and external) submitted to the Veterinary Diagnostic Laboratory at UCD was searched for pathology reports from dogs that included the words “thyroid” from January 2007 to January 2019. In addition to the three cases described above, thyroid cystadenomas were diagnosed in three further dogs that died from unrelated two of which were boxer dogs diseases, two of which were boxer dogs (Table 3). Clinicopathological information was limited for these external cases. One of the boxers (U351634) was an entire male diagnosed post-mortem with unilateral right cystadenoma with the cyst measuring approximately 1 cm in diameter. The dog died because of a large aortic body carcinoma with marked infiltration of the right atrium. The other boxer (U243968) was also an entire male and was diagnosed post-mortem with two large cysts occurring in the left thyroid gland (Fig. 5). The dog died as a result of severe diffuse bilateral renal fibrosis. Unfortunately, further information regarding thyroid function of these two external cases could not be retrieved. In addition, a cyst (2.5 cm × 1.3 cm × 1 cm) was reported in association with a bilateral follicular thyroid carcinoma in a 12-year-old bichon frise.

**Table 3:**
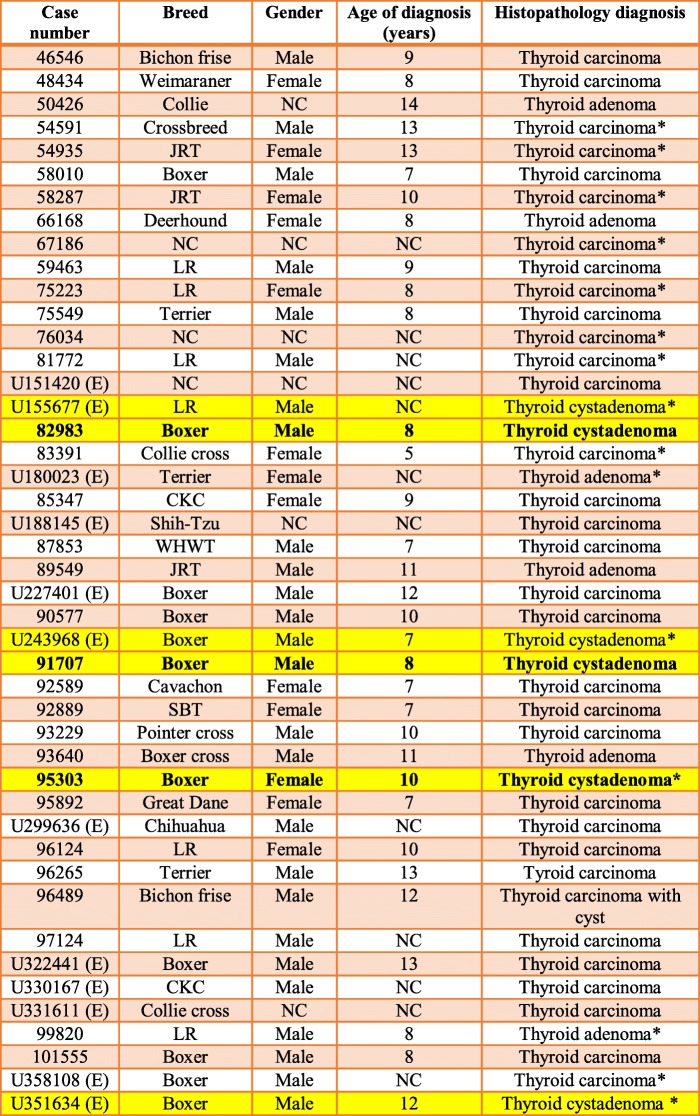
Cases of thyroid tumours in dogs identified through a database search of the UCD Veterinary Diagnostic Laboratory records from January 2007 to January 2019. The highlighted cases are the thyroid cystadenoma cases, with in bold the three cases described in this report

NC: Not communicated;

(E): External sample;

*Diagnosis at post-mortem;

*LR* Labrador retriever;

*JRT* Jack Russell terrier;

*CKC* Cavalier King Charles;

*WHWT* West Highland White terrier;

*SBT* Staffordshire bull terrier

## Discussion

The current case report describes the presence of an uncommon tumour, thyroid cystadenoma, in three boxer dogs. Although this tumour type has been previously reported in dogs, detailed clinical features are lacking [1, 5, 7, 18]. Greater detail is provided in two case reports, but the clinical appearance differed substantially from the current cases because of the presence of hyperthyroidism [19, 20]. In the current study, investigation was prompted by identification of a mass, or clinical signs secondary to a mass, rather than the consequences of hyperthyroidism. In addition, the presence of this tumour type in three dogs of the same breed may suggest a possible breed predisposition; a finding that is further supported by the identification of this tumour type mainly in boxer dogs in the database review.

Non-functional benign thyroid masses can cause clinical signs because of their size and localisation. Dysphagia, dyspnoea, cough or regurgitation can occur because of compression of surrounding tissues such as the trachea and oesophagus. In case 1, regurgitation and increased respiratory effort after strenuous exercise and excitement were reported. In this case, the CT of the neck and chest showed that the mass was causing intermittent partial obstruction of the tracheal lumen by compression of the tracheal membrane. Case 3 was reported to drool excessively. Although potentially related to the thyroid masses, the subsequent discovery of a rapidly growing oral neoplasm was considered a more likely cause.

None of the dogs had overt clinical signs of hypothyroidism at the time of diagnosis. This is not unexpected with unilateral lesions. However, information on thyroid function was not available for Case 1 and thyroid dysfunction could not be definitively ruled out. Case 2 had a low total T4 with upper reference interval cTSH concentration. This may suggest subclinical thyroid dysfunction given that primary hypothyroidism was subsequently definitively diagnosed six weeks after removal of the thyroid cystadenoma. Neoplasia within, and subsequent removal of, the first lobe may have contributed to decreased functional thyroid reserve and the development of overt hypothyroidism. In this case, the T4 concentration of the cystic fluid was lower compared to cases 1 and 3. This raises the possibility that the T4 concentration in the cystic fluid may provide some information on thyroid function that warrants further investigation. In humans, post-operative hypothyroidism has been reported in up to one third of patients following hemithyroidectomy because of suspected neoplasia [21–23]. In these cases, lymphocytic infiltration was identified within the resected tissue, but it is unclear if the tumour itself acts as a trigger for autoimmune thyroiditis [21] or if this represents concurrent but unrelated immune-mediated disease [22]. Lymphocytic infiltration was not apparent histologically in the resected tissue of any of the current cases, but thyroglobulin autoantibodies were not assessed. Therefore, the cause of hypothyroidism in case 2 remains unclear as the contralateral thyroid lobe was not removed or sampled.

It has been proven that fine needle aspiration (FNA) of solid cervical masses has a good diagnostic accuracy in humans [24]. In dogs, one study showed that correlation between cytological results and histopathological findings for thyroid carcinomas was only fair and the aspiration technique resulted in excessive contamination with blood in one third of cases [25]. Cytology was performed in all cases and only the FNA results in case 3 were consistent with the histopathologic diagnosis of adenoma. It is believed that this sample included tissue from the solid part of the thyroid tumour, as the enlargement was not confined to a single cyst in this case. In cases 1 and 2, cytologic evaluation was made from the aspirated fluid and was not diagnostic. This supports previous reports in which cytology of cystic fluid has rarely offered evidence of a particular tumour type or origin due to the paucity of cellular material and lack of pathognomonic cytological features, revealing only cell necrosis and blood cells [19, 20].

In adult humans presenting with a solitary cystic mass in the lateral neck, measurement of thyroglobulin concentration in the cystic fluid is advised as an initial step to confirm the thyroidal origin [27, 28]. Canine thyroglobulin assays are not commercially available. However, a similar approach was taken by measurement of cystic T4 concentrations, and the increased values rapidly confirmed thyroidal origin in all three cases. This appears to be a useful diagnostic procedure in dogs but has only been reported once before [19].

Although long-term effects of thyroid cysts are not known, drainage was only temporarily beneficial. Without treatment, thyroid cysts could continue to grow with progressive compression of adjacent structures, or malignant transformation from adenoma to carcinoma could occur. Although only suspected in dogs, this has been suggested to occur in cats with hyperthyroidism [29]. Also, one study suggested that in theory, TSH may contribute to further growth of primary thyroid carcinomas in dogs [30]. Surgical excision was therefore recommended and curative in the two cases in which it was performed. In humans, thyroidectomy is recommended for all patients with thyroid cysts who have a history of prior neck irradiation, abnormal FNA cytology results, recurrence of the cyst despite two or more needle aspirations and compressive clinical signs despite draining the cystic fluid [31].

In small animals, thyroid cystadenomas are described as follicular cell adenomas with one or two large cavities filled with proteinaceous fluid, necrotic debris and erythrocytes [7, 15, 17]. Cysts develop due to follicular distension and degeneration within thyroid tumours [17]. Further enlargement occurs due to frequent haemorrhage and desquamation of the follicular cells into the cystic lumen [15]. Follicular cysts have also been described in which the fluid filled cavities are lined by a dense fibrous capsule from which fronds of uniform cells arranged in follicular and/or compact cellular patterns project [8]. In these cases, neoplastic tissue is not identified. However, follicular cysts show similar morphological features to cystadenomas, and the differentiation can be somewhat arbitrary and dependent upon whether or not solid adenomatous tissue is present within the section examined [17, 24, 32]. Indeed, adenomatous change was not initially observed in case 2, and was only identified after preparation and examination of additional histopathologic sections.

Interestingly, all three dogs in the present study were boxers; a breed in which thyroid tumours have been reported with increased frequency in some [2, 7, 8] but not all [1] previous studies. The UCD Veterinary Diagnostic Laboratory database search of all internal and external samples over a 12-year period revealed the presence of this tumour type in only six cases (including the three dogs included in this case report), and five of these were boxer dogs. Whether boxers are truly predisposed to this particular condition warrants further investigation.

To the authors’ knowledge, this is the first report of non-functional thyroid cystadenomas in dogs. The cystic thyroid structures were detectable clinically because of their large volume but were not associated with clinical signs related to thyroid dysfunction. Measurement of T4 concentration in the cystic fluid was supportive of thyroidal origin and should be considered as a simple, relatively inexpensive first line diagnostic tool. Despite their apparent rarity, thyroid cystadenoma should be considered a differential for dogs presenting with cervical masses. Surgical excision is recommended as it is curative and because it provides tissue samples to investigate the underlying thyroid lesion.

## Data Availability

All data generated during this study are included in this published article.

## Abbreviations

CT: Computed tomographycTSH: canine thyroid-stimulating hormone
FNA: Fine needle aspiration
RBC: Red blood cells
T4: Thyroxine
UCD: University College Dublin
